# Inter- and intra-limb coordination during initial sprint acceleration

**DOI:** 10.1242/bio.059501

**Published:** 2022-10-03

**Authors:** Byron J. Donaldson, Neil E. Bezodis, Helen Bayne

**Affiliations:** ^1^ Sport, Exercise Medicine and Lifestyle Institute (SEMLI) and Department of Physiology, Faculty of Health Sciences, University of Pretoria, Pretoria, 0002, South Africa; ^2^ Applied Sports, Technology, Exercise and Medicine (A-STEM) Research Centre, Department of Sport and Exercise Sciences, Swansea University, Swansea, SA1 8EN, UK

**Keywords:** Dynamical systems, Kinematics, Segment dominancy, Sprinting, Technique

## Abstract

In complex movements, centre of mass translation is achieved through effective joint and segment rotations. Understanding segment organisation and coordination is therefore paramount to understanding technique. This study sought to comprehensively describe inter- and intra-limb coordination and assess step-to-step changes and between-individual variation in coordination during initial sprint acceleration. Twenty-one highly trained to world class male (100 m PB 9.89-11.15 s) and female (100 m PB:11.46-12.14 s) sprinters completed sprint trials of at least 20 m from which sagittal plane kinematics were obtained for the first four steps using inertial measurement units (200 Hz). Thigh-thigh, trunk-shank and shank-foot coordination was assessed using a modified vector coding and segment dominancy approach. Common coordination patterns emerged for all segment couplings across sexes and performance levels, suggesting strong task constraints. Between-individual variation in inter-limb thigh coordination was highest in early flight, while trunk-shank and shank-foot variation was highest in late flight, with a second peak in late stance for the trunk-shank coupling. There were clear step-to-step changes in coordination, with step 1 being distinctly different to subsequent steps. The results demonstrate that inter-limb coordination is primarily anti-phase and trailing leg dominant while ankle motion in flight and late stance appears to be primarily driven by the foot.

## Teaser

**Summary:** There are common inter- and intra-limb coordination patterns across well-trained sprinters with step-to-step changes within individuals during the first steps of sprint acceleration.

## INTRODUCTION

Acceleration from a stationary block position in athletic sprinting is a complex task with important implications for race performance (Bezodis et al., 2015; Bezodis et al., 2019b; Walker et al., 2021). Initial acceleration consists of the first 3-6 steps after block exit and is distinguished from later phases by step-to-step kinematic changes (Nagahara et al., 2014; von Lieres und Wilkau et al., 2018). As such, researchers and coaches approach initial acceleration as a unique technical component of the sprint (e.g. Debaere et al., 2013; Bezodis et al., 2019a; Walker et al., 2021; Jones et al., 2009). Effective acceleration depends more on force vector orientation than total magnitude of the resultant force generated (Morin et al., 2011; Kugler and Janshen, 2010; Rabita et al., 2015), with more horizontally directed forces corresponding to a further forward centre of mass (CM) (Slawinski et al., 2010; Kugler and Janshen, 2010). Since CM position is a function of overall musculoskeletal system organisation, a horizontal CM position, and horizontal force application, results from effectively organising the linked segment system (Nagahara et al., 2014; von Lieres und Wilkau et al., 2018; Kugler and Janshen, 2010; Slawinski et al., 2010). Whilst existing literature has quantified isolated joint and segment kinematics during initial acceleration (e.g. Walker et al., 2021; von Lieres und Wilkau et al., 2018; Nagahara et al., 2014, 2018; Slawinski et al., 2010; Debaere et al., 2013), an evaluation of the relative movement these system components is needed to understand how sprinters coordinate the motion of their available degrees of freedom to satisfy the task constraints (Glazier, 2017; Davids et al., 2003; Newell, 1986). By quantifying this in a cohort of highly skilled sprinters, the importance of both organismic and task constraints can be understood by assessing the aspects of emergent coordination patterns unique to individuals (organismic) and the similarity of coordination patterns between individuals (task) during maximal sprint acceleration efforts.

In linear sprinting, the vast majority of movement is in the sagittal plane, and therefore most research and coaching analyses of segment kinematics are focused on sagittal plane trunk and lower limb motion (e.g. Nagahara et al., 2014, 2018; Debaere et al., 2013; Clark et al., 2020). During initial acceleration, there is a step-to-step raising of the CM in part due to step-to-step shifts toward more vertical shank and trunk segments (Nagahara et al., 2014; von Lieres und Wilkau et al., 2018). Better performers exhibit smaller shifts towards a vertical trunk orientation over the initial steps (von Lieres und Wilkau et al., 2018; Kugler and Janshen, 2010) while a more horizontal trunk at toe off is associated with better performance during the first stance of world class sprinters (Walker et al., 2021). However, as a more proximal segment, trunk motion during stance could be a function of more distal (stance leg) segment orientations. The trunk typically rotates clockwise (as viewed from the right) during flight before reversing direction during stance (Nagahara et al., 2018; Donaldson et al., 2020), whilst the shank does the opposite - rotating anticlockwise toward a vertical orientation during flight and the opposite during stance, rotating back toward a horizontal orientation (Nagahara et al., 2014; von Lieres und Wilkau et al., 2018; Donaldson et al., 2020). However, the relationship between the timing and relative magnitude of these rotations is unclear. Given the coaching interest in the relationship between trunk and shank angles at key events (Donaldson et al., 2020; von Lieres und Wilkau et al., 2018) and the role both the shank and the trunk play in facilitating more forward CM positions and orienting force in the horizontal direction (Kugler and Janshen, 2010; Jacobs and van Ingen Schenau, 1992; von Lieres und Wilkau et al., 2018; Alt et al., 2022), understanding of this inter-segmental relationship is needed. In the only study to so far investigate trunk-shank coordination in sprinting, Bezodis et al. (2019a) found in-phase coordination (same rotation direction) during mid stance, suggesting timing differences in trunk and shank rotation reversals. Further understanding shank and trunk coordination may provide important insight regarding CM raising and forward translation during acceleration.

Several studies have established the importance of ankle energy absorption and power generation during acceleration (Bezodis et al., 2014; Gittoes and Wilson, 2010; Charalambous et al., 2012; Debaere et al., 2013), while ankle stiffness has been associated with acceleration performance (Charalambous et al., 2012). However, little is known about how ankle dorsiflexion and plantarflexion are achieved by the motion of the segments which comprise the joint. Indeed, no study has investigated shank and foot coordination, with a resultant gap in understanding of the relative contributions of shank and foot rotation to ankle motion. Theoretically, the changes in shank angle across acceleration (Nagahara et al., 2014; von Lieres und Wilkau et al., 2018) suggest possible changes to geometric constraints (van Ingen Schenau et al., 1987), which could alter the relative importance of shank or foot rotation to ankle motion in different steps. Studies of shank-foot coordination are required to elucidate the roles of the shank and foot to ankle motion within a step as well as the shift in their relationship between steps and phases.

Recent studies have investigated thigh angular motion during maximal velocity (Clark et al., 2020; Kakehata et al., 2021), acceleration (Bayne et al., 2020; Walker et al., 2021) and uphill sprinting (Okudaira et al., 2021). From coaching observations, such investigations focus on ‘switching’ and ‘scissoring’, the respective points where the thighs cross over or reverse rotation (Clark et al., 2020; Okudaira et al., 2021; Kakehata et al., 2021). Clark et al. (2020) found faster sprinters had greater thigh angular accelerations and greater average thigh angular velocity over the gait cycle, suggesting the ability to rapidly transition between thigh flexion and extension is important. During acceleration, maximal velocity and uphill running, the thighs produce an oscillatory motion rotating in opposing directions, with one flexing and the other extending (Clark et al., 2020; Bayne et al., 2020; Okudaira et al., 2021), resulting from unique constraints on human bipedal gait (Kiely and Collins, 2016). Only two studies have investigated inter-limb thigh coordination. Bayne et al. (2020) found elite sprinters spent more of the step in anti-phase (opposing rotation) than sub-elite counterparts during initial acceleration while Okudaira et al. (2021) found increased anti-phase with increased incline in uphill sprinting. However, neither study found anti-phase motion at all time points and were not able to identify why that was the case or any other characteristic features of thigh coordination. It also remains unclear whether oscillatory thigh motion is driven equally by each leg or if there is greater rotation in one leg at any given time.

To date, sprint coordination studies have primarily focused on coordination patterns between two groups over multiple steps, with less emphasis on potential differences between steps or individuals (Bayne et al., 2020; Okudaira et al., 2021; Bezodis et al., 2019a). However, previous literature suggests the first step may have unique characteristics (Charalambous et al., 2012; Bezodis et al., 2014) and key segment angles change from step-to-step during initial acceleration (Nagahara et al., 2014; von Lieres und Wilkau et al., 2018; Donaldson et al., 2020). It remains unclear whether there are concomitant differences between steps in segment coordination and what that might imply about the constraints on initial acceleration technique. Further, given the self-organising nature of coordination patterns, group-based analyses could overlook between-individual variation and make it harder to identify underlying causes and constraints from which technique differences between individuals may arise.

Understanding segment organisation is essential to understanding sprint technique. Considering the coaching emphasis on kinematics, the task's technical nature and step-to-step changes during initial acceleration, a comprehensive description of whole-body coordinative strategies is warranted and can provide unique insight into both task- and individual-related aspects of technique. Therefore, this study aimed to comprehensively describe relevant intra- and inter-limb coordination couples and segment dominancy during initial acceleration in highly trained to world-class male and female sprinters, and to quantify between-individual variation and step-to-step changes in these features.

## RESULTS AND DISCUSSION

This study aimed to comprehensively describe intra- and inter-limb coordination strategies in well trained sprinters and to quantify between-individual variation and step-to-step changes in coordination.

**Fig. 1.**
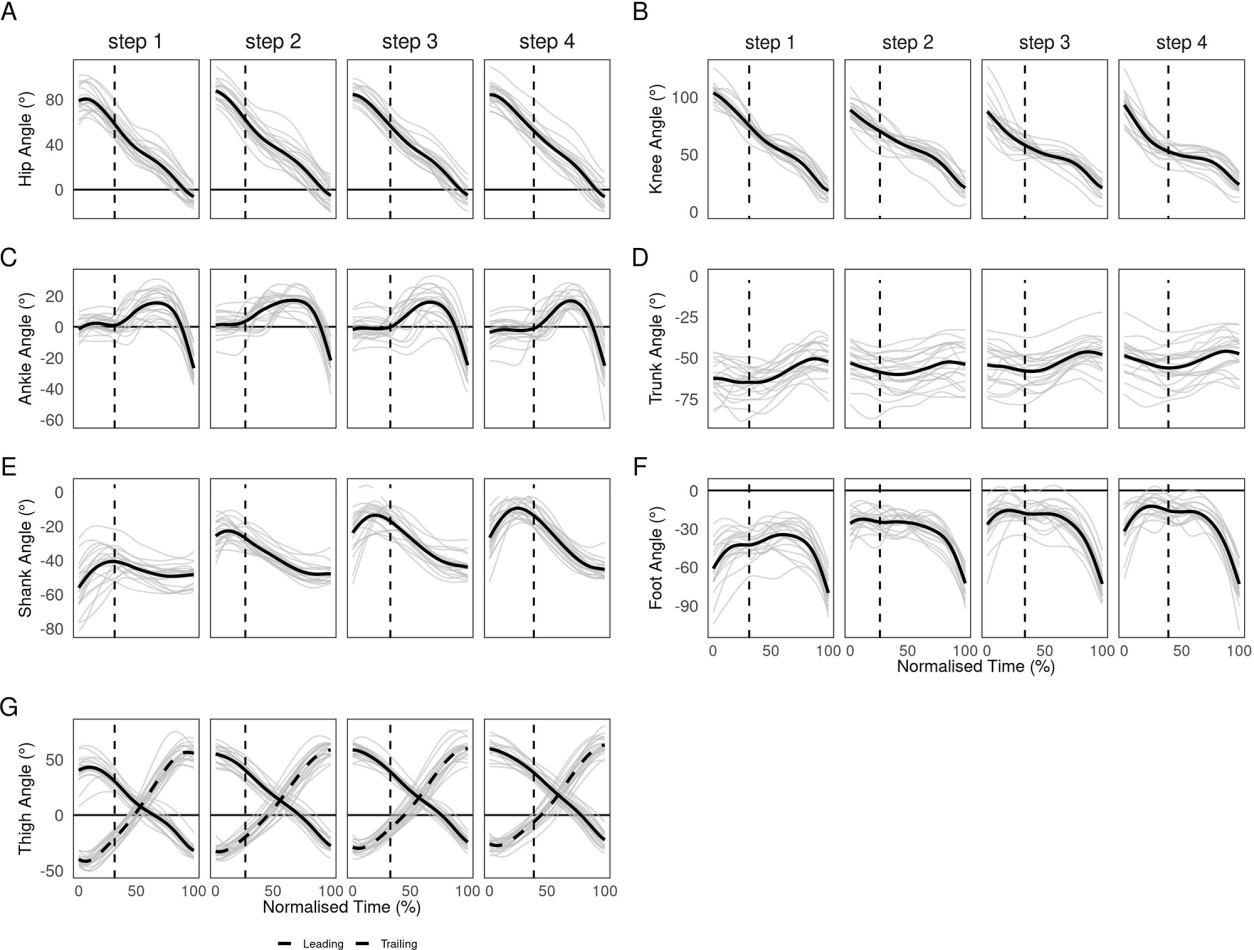
**Mean (black) and individual (grey) continuous hip (A), knee (B), ankle (C), trunk (D), shank (E), foot (F) and leading and trailing thigh (G) angles for each of the first four steps after block clearance (step 1, 0% time).** Vertical dotted line indicates mean touchdown time (%).

The current results agree with previous segment and joint kinematics investigations by Nagahara et al. (2014) and von Lieres und Wilkau et al. (2018), finding significant main effects of step for all segment and joint touchdown angles except the ankle, as well as trunk, thigh, shank and foot angles at toe off (Table 1, Fig. 1). However, particularly at touchdown, pairwise tests revealed step 1 to be different from all subsequent steps while step 2 commonly differed from both step 1 and later steps, with only the knee significantly different between steps 3 and 4. As such, step 1 and 2 touchdown kinematics were different from both each other and later steps. Both the trunk (*F*_(3,60)_=11.7, *p*<0.001, *η*^2^=0.37) and shank (*F*_(3,60)_=138.4, *p*<0.001, *η*^2^=0.874) became progressively more vertical at touchdown, however trunk angle only differed significantly between step 1 and all subsequent steps. In contrast, the foot contacted the ground in a more vertical orientation in step 1 and decreased progressively, with a sharp decrease between step 1 and 2 (*F*_(2.11,42.3)_=61.5, *p*<0.001, *η*^2^=0.754). Similarly, toe off foot angle was significantly more vertical in step 1 than later steps (*F*_(3,60)_=3.1, *p*=0.032, *η*^2^=0.136). The thigh was less flexed at touchdown in step1 compared to later steps (*F*_(3,60)_=10.2, *p*<0.001, *η*^2^=0.337) but more extended at toe off in step 1 and 2 compared to 3 and 4 (*F*_(3,60)_=24.7, *p*<0.001, *η*^2^=0.552).

**Table 1.**
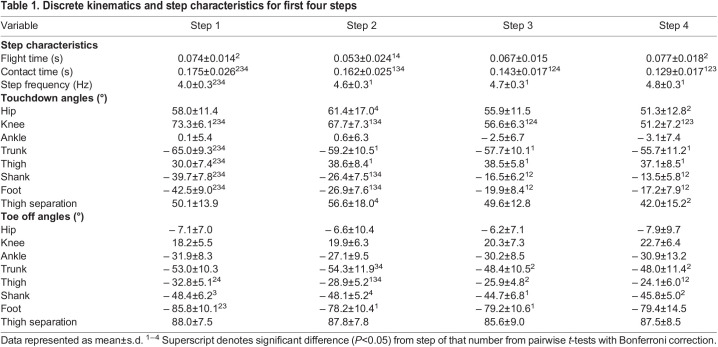
Discrete kinematics and step characteristics for first four steps

**Fig. 2.**
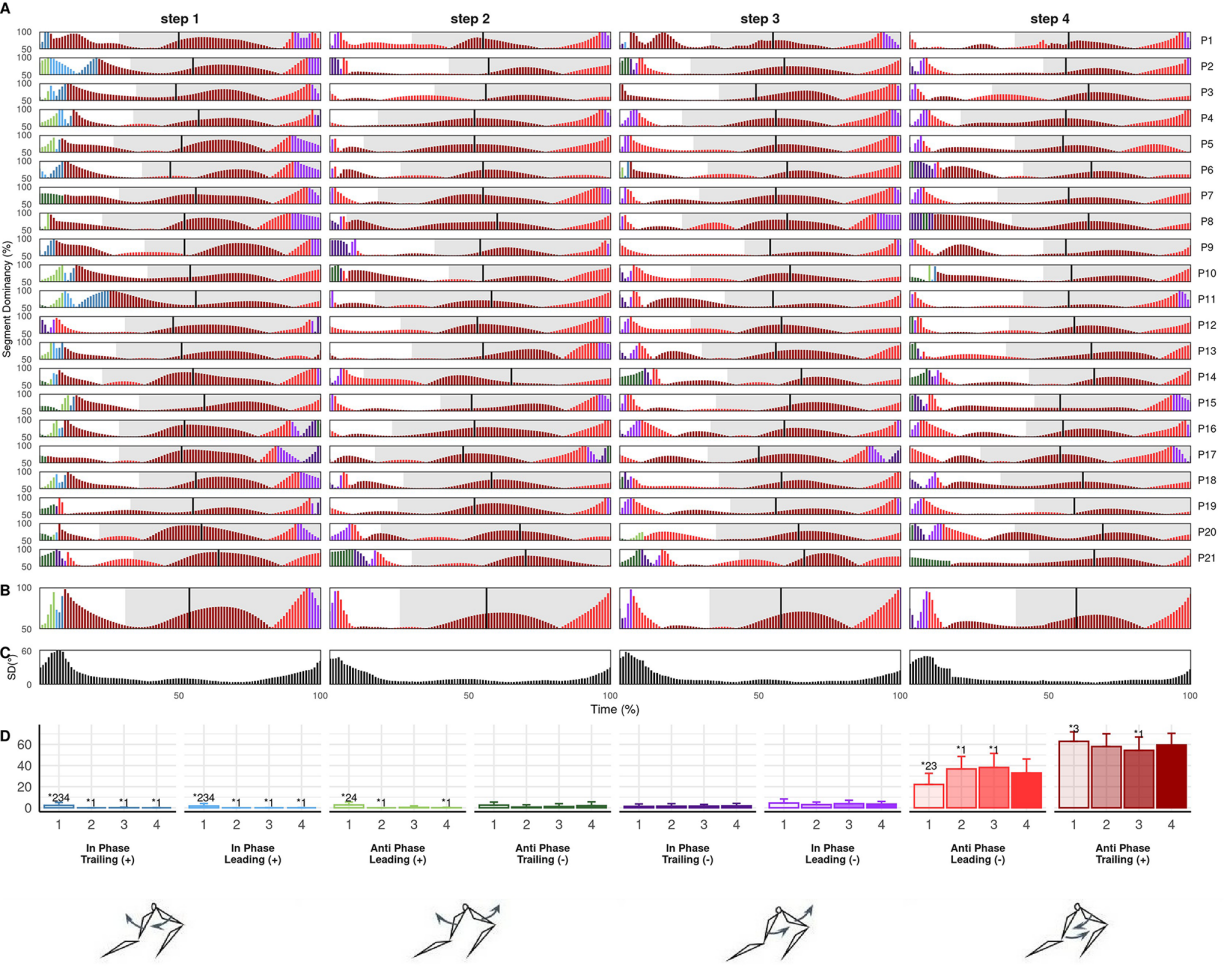
**Individual (A) and mean (B) thigh-thigh coordination profiles, between-individual standard deviation (C) and mean bin frequencies (D) across steps 1 to 4.** Coordination profile bar height shows segment dominancy (50-100%) and colour shows bin classification according to colour scale of bin frequency plot (D). Grey shaded area indicates stance, black vertical line indicates point of crossover.

**Fig. 3.**
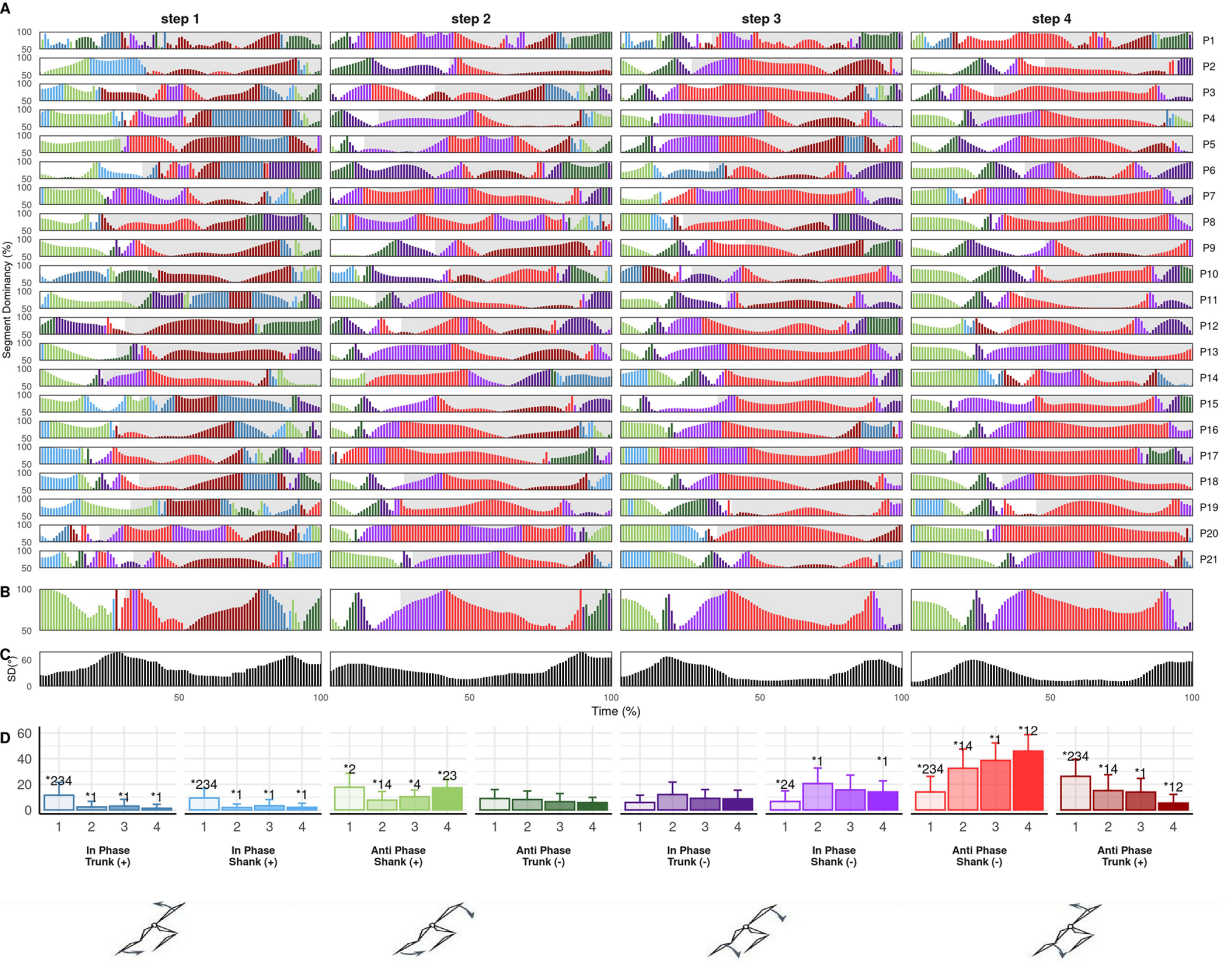
**Individual (A) and mean (B) trunk-shank coordination profiles, between-individual standard deviation (C) and mean bin frequencies (D) across steps 1 to 4.** Coordination profile bar height shows segment dominancy (50-100%) and colour shows bin classification according to colour scale of bin frequency plot (D). Grey shaded area indicates stance.

**Fig. 4.**
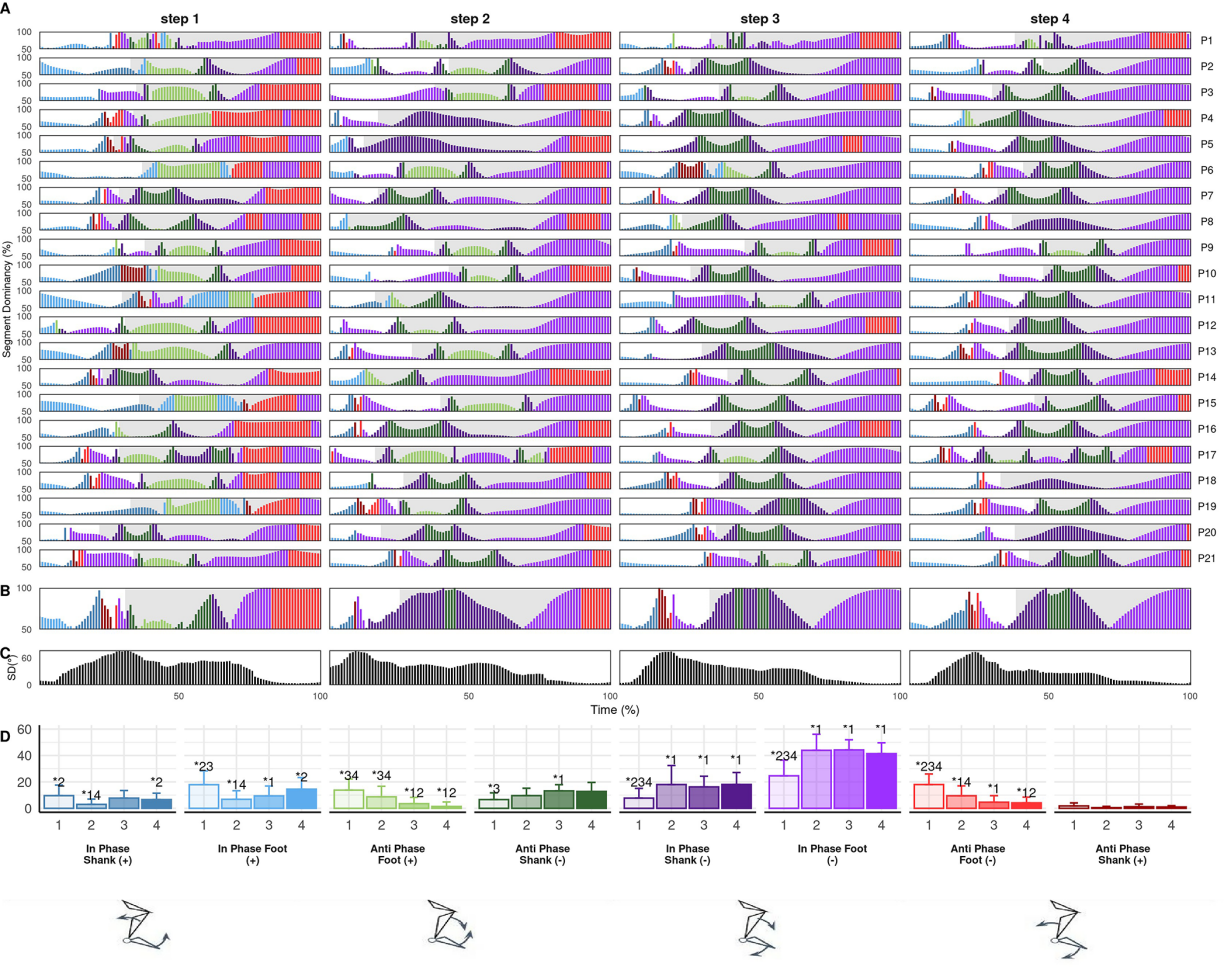
**Individual (A) and mean (B) shank-foot coordination profiles, between-individual standard deviation (C) and mean bin frequencies (D) across steps 1 to 4.** Coordination profile bar height shows segment dominancy (50-100%) and colour shows bin classification according to colour scale of bin frequency plot (D). Grey shaded area indicates stance.

There were large step-to-step coordination differences for all couplings, with similar between-step differences in trunk-shank (CA_Diff_: S1-S2=33.5±9.2%, S2-S3=22.8±9.8%, S3-S4=16.5±6.6%) and shank-foot coordination (CA_Diff_: S1-S2=29.7±11.3%, S2-S3=23.6±6.9%, S3-S4=16.9±7.2%) while thigh-thigh between-step differences were smaller (CA_Diff_: S1-S2=12.5±4.0%, S2-S3=9.0±3.3%, S3-S4=9.1±3.6%). In all cases, the largest differences were between steps 1 and 2, indicating between-step coordination became progressively more similar. Moreover, there were significant main effects of step on bin frequencies across all couplings, with the most common pattern being significant differences between step 1 and all three subsequent steps (Figs 2-4D). The step-to-step differences in both coordination and isolated kinematics suggest the first step is technically different from subsequent steps and the step 1 to 2 transition could be considered an additional breakpoint to the one previously identified around steps 3-6 (Nagahara et al., 2014; von Lieres und Wilkau et al., 2018), which supports the emphasis placed on the first step by elite coaches (Jones et al., 2009). Significant differences at touchdown and in certain coordination bins mean that step 2 could also be considered separately, although step 2 differences from later steps were less consistent than step at touchdown and there were fewer differences in coordination. Coordination differences between step 1 and later steps most likely reflects the unique constraints of block exit in athletic sprinting and may exhibit smaller differences when accelerating from other start positions.

### Thigh-thigh coordination

Thigh-thigh coordination was primarily anti-phase and trailing thigh dominant (dark red) (Fig. 2), reflecting the oscillatory motion associated with bipedal gait (Kiely and Collins, 2016) and supporting the high frequency of anti-phase thigh coordination that Bayne et al. (2020) and Okudaira et al. (2021), respectively, reported during acceleration and uphill sprinting. The oscillatory anti-phase thigh motion also aligned with thigh motion reported by Clark and colleagues during maximal velocity sprinting (Clark et al., 2020) (Fig. 1G), suggesting that thigh angular motion may already be similar to that of maximal velocity sprinting within the first few steps. However, there was substantial trailing leg dominance, such that oscillatory thigh motion appears asymmetric and characterised by faster forward rotation of the trail leg during acceleration. The mean frequency of anti-phase trailing (+) (dark red) decreased progressively from step 1 to step 3 and was significantly higher in step 1 (73) than step 3 (54%, *P*=0.024). Trailing leg dominance was typically highest at or shortly after crossover between the two thighs (see Fig. 2A,B, black vertical line). The magnitude of trailing leg dominance around crossover was highest in step 1, reaching almost 100% dominance in some cases, and this decreased progressively in subsequent steps. Thigh-thigh coordination variation was highest in early flight, but low for the majority of the step before a gradual increase in late stance. Thus, variation in coordination strategy was highest around block clearance and toe off, owing to the respective timing of flexion and extension reversals during the scissor action. Generally low variation in thigh-thigh coordination implies strong task constraints for inter-limb coordination in acceleration, with greater degrees of freedom potentially available to athletes during transitions between steps. Thigh coordination was visibly different after block clearance compared to after toe off in subsequent steps. Most participants exhibited anti-phase leading (+) (light green) or trailing (−) (dark green) coordination immediately after block clearance, reflecting trailing thigh rotating clockwise and leading thigh anti-clockwise, increasing thigh separation. Further, approximately half of participants displayed a short in-phase (+) (light and dark blue) period (Fig. 2A), with these observations reflected in significantly higher frequencies of anti-phase leading (+) and in-phase (+) bins compared to later steps (Fig. 2D). Thus, some athletes may require time in the first flight to sufficiently organise the limbs before initiating the scissor action, particularly for the lead leg. Whether additional time is beneficial or inefficient remains unclear. The in-phase period (light and dark blue) demonstrates an asymmetric scissor after block exit, starting to pull the trail leg forward slightly before starting to retract the lead leg. Continued lead leg anticlockwise rotation after block clearance could reflect an athlete's intent to achieve maximum drive out the blocks or it could indicate that an athlete has not been able to bring the lead leg forward to its full extent before the front leg exits the blocks. Further investigations might determine whether the observed patterns reflect inefficiencies in thigh organisation at block clearance, specific organismic constraints or necessary task constraints associated with exiting the blocks.

In later steps, there were three patterns after toe off. A minority of participants displayed anti-phase trailing (-) (dark green), the same continued increase of thigh separation seen after block clearance but more trail leg dominant (for example Fig. 2A, P21). These participants did not start to pull the trail leg forward or retract the lead leg until after toe off. Therefore, such an anti-phase pattern might indicate a cyclic leg action since the lead leg does not retract immediately at toe off but continues anticlockwise rotation during the initial flight, possibly inhibiting an athlete's ability to execute cues to ‘aggressively switch’ or ‘hammer’ the ground, used by some coaches to emphasise aggressive leg retraction during the scissor action and into the next ground contact. Most participants exhibited either anti-phase (light and dark red) or in-phase (−) (light and dark purple), where anti-phase showed the scissor had already happened and in-phase indicated both legs rotating clockwise. In-phase coordination in initial flight shows a late switch in trail leg rotation and could be suggestive of what some coaches label ‘over pushing’. The individual characteristics that lead an athlete to adopt this pattern and what the implications for performance might be remains unclear.

In late stance, participants either continued anti-phase rotation until toe off or they displayed in-phase leading (-) (light purple) (Fig. 2) coordination. In-phase motion was most common in step 1 and reduced in both occurrence (number of athletes) and proportion (% of step) in later steps, a pattern corresponding with greater stance thigh angles at toe off in step 1 (Table 1). In-phase coordination represents a swing thigh (trail thigh at this stage) reaching an earlier maximum angle relative to the stance thigh and beginning to retract before toe off, and therefore an asymmetric scissor. Walker et al. (2021) found greater thigh separation at step 1 was associated with greater external horizontal power and postulated that this might be the way athletes maximise thigh angular velocity of the retracting thigh in the next step. Thigh separation angles at toe off were smaller in the current study than Walker et al. (2021), and the in-phase coordination present in late stance implies maximum thigh separation occurs prior to toe off for many participants, especially in step 1. No participant exhibited a perfect scissor, i.e. continuous anti-phase leading (-) (light red) or trailing (+) (dark red) across toe off, with all requiring some in-phase (-) or anti-phase trailing (-) in either late stance or early flight. Such patterns may be necessary to facilitate the scissor action during acceleration or may indicate than none of the current cohort were able to exhibit a fully sound technical strategy. No studies of inter-limb coordination in maximal velocity sprinting exist, so it remains possible that a perfect scissor action can be achieved in later phases but because of short flight times, long contact times and the asymmetrical push from the blocks during initial acceleration, perfect scissoring is not possible in the first few steps. Future research may determine whether in-phase coordination around toe off is necessary or represents inefficiencies in scissor execution.

### Trunk-shank coordination

**Fig. 6.**
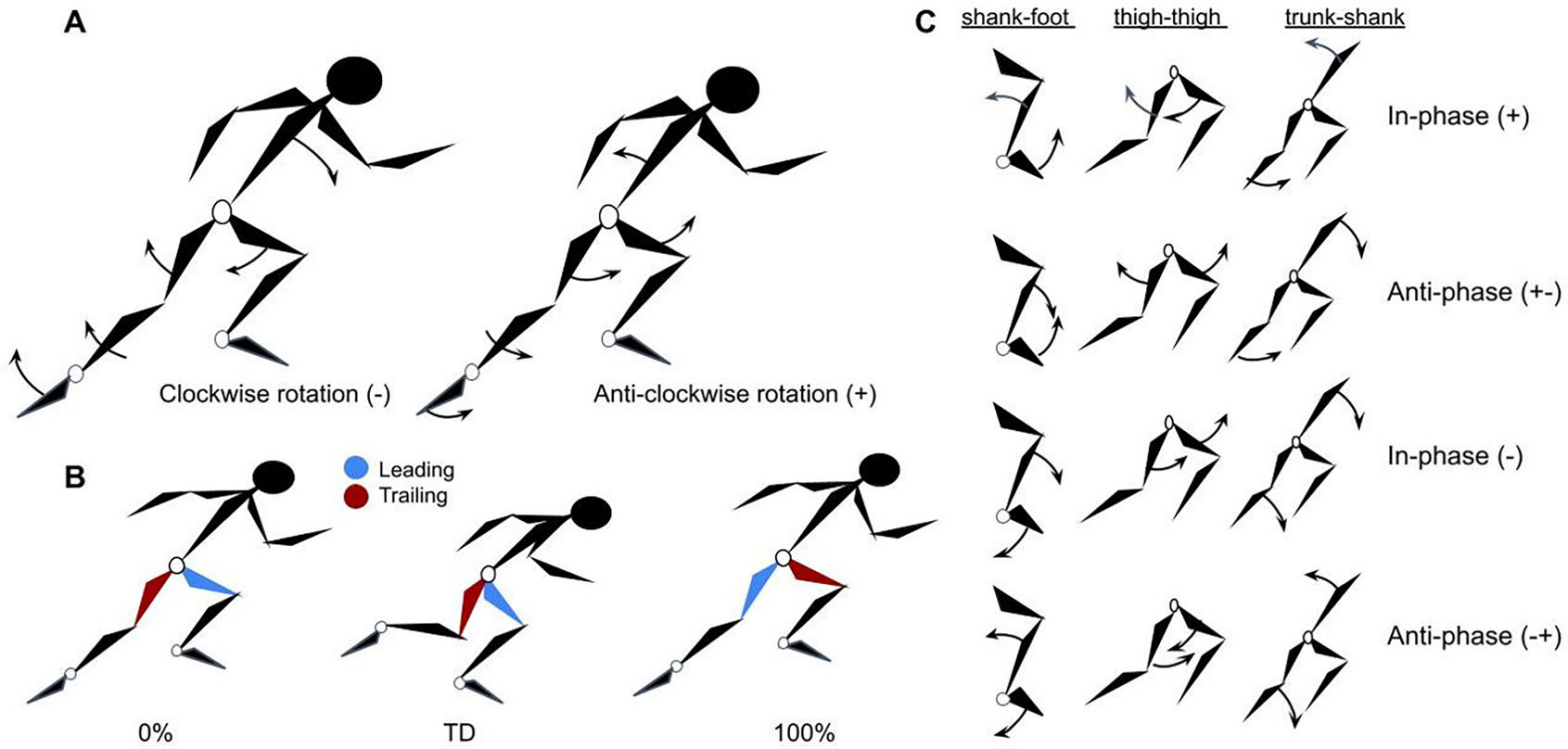
Segment rotation (A) and leading leg-trailing leg (B) conventions with coordination direction of rotations (C).

Trunk-shank coordination was mostly shank dominant, demonstrating relatively greater shank than trunk rotation over the step cycle, and the frequency of shank dominant coordination, especially during stance, increased significantly from step-to-step (Fig. 3A,D). However there was prolonged trunk dominance during stance in step 1, with step 1 anti-phase trunk (+) (dark red) and in-phase trunk (+) (dark blue) bin frequencies significantly higher than later steps (Fig. 3A,D). This likely resulted from more horizontal shank and trunk orientations at block clearance and touchdown in step 1 compared to later steps, producing less clockwise shank rotation and more anticlockwise trunk rotation, potentially indicating specific task constraints associated with block exit (Table 1, Fig. 3). These results agree with Bezodis and colleagues' analysis of the first and third stance, where coordination defined by trunk rotation in mid and late stance in step 1 was absent in step 3 (Bezodis et al., 2019a). In flight, trunk-shank coordination was anti-phase shank (+) (light green), reflecting clockwise trunk rotation towards the horizontal and anticlockwise shank rotation toward the vertical. From step 2 onwards, there was commonly in-phase shank (−) (light purple) around touchdown before becoming predominantly anti-phase shank (−) (light red) (Fig. 6). Therefore, typical coordination patterns were clockwise trunk and anticlockwise shank rotation during flight and the reverse during stance, in accordance with previously reported trunk and shank motion (Nagahara et al., 2018, 2014; Donaldson et al., 2020; von Lieres und Wilkau et al., 2018). However, in-phase coordination around touchdown reveals novel insight into the relative timing of reversals; the trunk switches direction later than the shank, yielding simultaneous clockwise rotation around touchdown and early stance. Jacobs and van Ingen Schenau (1992) theorised a stereotyped action in sprinters where the CM, through what Alt et al. (2022) have called ‘shin roll’, achieves forward translation by first rotating over the point of ground contact and then through extension of the lower limb. The current results are consistent with that observation, such that the trunk and shank rotate forward in-phase during Jacobs and Van Ingen Schenau's (1992) rotation stage, before the trunk changes direction of rotation during the subsequent extension stage. Despite differences in coordination bin demarcation, overall trunk-shank coordination patterns observed here are similar to those Bezodis et al. (2019a) reported. Participants displayed clockwise trunk rotation again in late stance, while shank rotation was reduced (Fig. 3A,B), producing in-phase trunk (-) (dark purple) or anti-phase trunk (-) (dark green). Thus, the trunk and shank converge on the fully extended toe off body position sometimes discussed by coaches (Jones et al., 2009) from opposing directions, reaching similar toe off angles (Table 1). Thus, through the influence of trunk angle on CM position and the role of the shank in rotating the CM and directing the angle at which the more proximal segments extend, (Jacobs and van Ingen Schenau, 1992; Alt et al., 2022) these two segments appear to work in consort and may help to influence the direction of force output and CM motion at toe off. However, the shank achieves the desired toe off position relatively earlier than the trunk. The trunk then adjusts further in late stance, possibly already anticipating clockwise rotation in the next step. Between-individual variation was generally higher in trunk-shank compared to thigh-thigh coordination, with standard deviations reaching as much as 80° (Fig. 3C), likely due to trunk rotation highly variable in both the magnitude and direction across participants (Fig. 1D). Variation was highest in late flight and rose again in late stance. Therefore, variation in trunk-shank coordination increased prior to events in the gait cycle, suggesting athletes might adjust these segments in preparation for achieving the desired body positions at touchdown and toe off. Specific positions at these events might present challenges that athletes approach in different ways due to varying organismic constraints.

### Shank-foot coordination

Several common shank-foot patterns emerged across participants, which may stem from sub-groups of athletes with similar constraints, while there was high between-individual variation - primarily due to differences in timing of common coordination features around late flight and early stance (Fig. 4A-C) and variation in foot angle (Fig. 1F). Coordination in flight was in-phase foot (+) (light blue) which transitioned to in-phase shank (+) (dark blue). Given the conjoined nature the segments, such in-phase motion during flight may be expected. Before touchdown, particularly in steps 2 to 4, the shank and foot reversed to in-phase foot (−) (light purple) in preparation for ground contact. A key feature of shank-foot coordination surrounded ankle dorsiflexion during early stance. Dorsiflexion was characterised by anti-phase foot (+) (light green) and shank (−) (dark green) coordination, typically sandwiched by short in-phase shank (−) (dark purple) periods (Fig. 4A). In dorsiflexion, anti-clockwise foot rotation toward a flat orientation was coupled with clockwise shank rotation over the foot. Segment dominance during dorsiflexion differed between steps 1 and 2 compared to later steps. In step 1, dorsiflexion was associated with foot dominant anti-phase (light green) (Fig. 4), which remained true for approximately half of participants in step 2, with significantly more anti-phase foot (+) in steps 1 and 2 compared to steps 3 and 4 (Fig. 4D). Shank dominant dorsiflexion characterised these latter steps. Thus ankle dorsiflexion in initial stance (Charalambous et al., 2012; Bezodis et al., 2014; Bezodis et al., 2019b) is primarily driven by foot rotation in the first step and shifts to be shank driven in later steps, with a larger role for ‘shin roll’ (Alt et al., 2022) in later steps. The segment dominancy change reflects a transition from more horizontal shank and vertical foot orientations in step 1 towards more vertical shank and flat foot orientations in subsequent steps (Table 1, Fig. 1E,F), as well as less ankle dorsiflexion in earlier steps (Fig. 1C). More horizontal shank orientations in step 1 may require greater relative foot rotation to facilitate the energy absorption performed by ankle dorsiflexion in early stance (Bezodis et al., 2014), while a more vertical shank and flat foot at touchdown in later steps might require shank dominant coordination for ankle dorsiflexion and forward CM translation.

All participants exhibited dominant clockwise foot rotation [in-phase (light purple) and anti-phase (light red)] from mid stance onwards, with the magnitude of dominance increasing progressively (Fig. 4A,B). Step 1 had significantly more anti-phase foot (-) (light red) compared to later steps, with the frequency decreasing progressively over steps (Fig. 4D). Anti-phase prominence in the first step may result from geometric constraints (van Ingen Schenau et al., 1987; van Ingen Schenau, 1989) imposed by a more horizontal touchdown shank orientation, whereby the athlete cannot produce further shank rotation without compromising balance and therefore requires foot dominant action to translate the CM forward. Indeed, foot angle was significantly more vertical at toe off (Table 1) and the shank approached toe off angle earlier in step 1 than later steps, supporting the notion that greater foot rotation contributed to the forward CM translation which is an inherent feature of acceleration. A stable shank angle during late stance may help to enable foot rotation to yield forward CM translation. In subsequent steps, coordination tended to be in-phase, indicative of increasingly vertical shank and flat foot orientations at touchdown and less vertical foot at toe off. Yet, foot dominant in-phase rotation implies that even with a more vertical shank at touchdown, after the shank rotates over the foot during dorsiflexion, subsequent shank rotation observed during stance is driven by foot rotation. The progressive increase in magnitude of foot dominance suggests a key role for the foot in driving CM translation during initial acceleration, in accordance with the rotate and extend model of Jacobs and van Ingen Schenau (1992) and through the ‘shin roll’ concept developed by Alt et al. (2022). The initial rotation over the foot corresponds to the initial dorsiflexion observed in early stance, (Charalambous et al., 2012; Bezodis et al., 2014, 2019b) which is primarily shank dominant, but the subsequent ankle plantarflexion that occurs with the proximal-to-distal pattern of joint extension (Jacobs and van Ingen Schenau, 1992; Charalambous et al., 2012; Bezodis et al., 2014, 2019b) is driven by foot dominant rotation. Thus, the rapid ankle plantarflexion which translates the CM forward in late stance is almost entirely driven by clockwise foot rotation. The consistency of this pattern across participants highlights potentially strong constraints on available shank-foot coordination strategies during stance.

### Implications

While isolated segment kinematics have been well studied, the current study makes several novel contributions to understanding the relationships between segments, the changes in those relationships from step-to-step and the possible constraints on segment coordination during initial acceleration. These results indicate that initial sprint acceleration has relatively strong task constraints which yield broadly similar coordination patterns across a sample of male and female sprinters, including highly trained and world-class athletes. Furthermore, these task constraints do not appear consistent across all four steps following block exit. While previous studies of step-to-step kinematics identified steps 3-6 as a breakpoint in acceleration (Nagahara et al., 2014; von Lieres und Wilkau et al., 2018), others have suggested the first stance should be considered separately from later steps and phases (Charalambous et al., 2012; Bezodis et al., 2014). The current study supports the latter assertion, indicating that across thigh-thigh, trunk-shank and shank foot couplings, step 1 has unique coordination. Block exit appears to impose constraints on the first step that result in athletes adopting different coordination strategies compared to subsequent steps. Specifically, athletes seem to require relatively longer to organise the thigh segments after block exit than after toe off in later steps. Moreover, more horizontal trunk and shank orientations and more vertical foot placement at touchdown in step 1 result in more foot dominant shank-foot coordination and more trunk dominant trunk-shank coordination than steps 2-4. Foot dominant coordination from mid stance across all four steps implies a key role for the foot in driving CM translation during sprint acceleration, which might have implications for performance related factors like horizontal force application. Finally, there was a novel finding of substantial trail leg dominance in thigh-thigh coordination, highlighting asymmetric thigh rotation during acceleration with faster swing leg rotation. Between-individual variation was highest around touchdown and toe off, suggesting the main differences between individuals is how they prepare for, and respond to, these events as well as the relative timing in movement transitions associated with them. In particular, individuals' thigh-thigh coordination differed mostly in relation to toe off and the timing of reversals in thigh rotation. Shank-foot coordination, in contrast, was mostly different around touchdown and early stance. Understanding the potential individual constraints (strength, anatomy, stature, etc.), which may contribute to these differences should be the focus of future work. Further investigation is also required to determine whether different coordination strategies may be used to achieve the same performance outcome or whether better performance outcomes align with particular coordination approaches. Assessing the performance and physical capacities of athletes with similar coordination strategies may facilitate such an understanding.

### Conclusion

This study comprehensively described and quantified coordination during initial acceleration across a range of well-trained sprinters, identifying both common coordination patterns across the group as well as novel segment dominancy patterns in key relationships. Clear step to step changes in segment organisation and coordination were identified, with unique patterns observed in step 1. There are common coordination patterns amongst trained sprinters related to the task of accelerating, however, individualised profiling highlighted potential individual-specific strategies, particularly in preparation for, or as a result of, touchdown and toe off events. Inter-limb thigh coordination is primarily an anti-phase motion dominated by the trailing leg, while there is clear foot-dominance in shank-foot coordination in flight and late stance during acceleration, which may suggest an important role of the foot in intra-limb coordination strategies during acceleration.

## MATERIALS AND METHODS

### Participants

Fifteen male (age=22.0±3.6 years, stature=1.77±0.06 m, mass=74.6±9.7 kg, 100 m PB=10.47±0.42 s) and six female (age=22.8±6.5 years, stature=1.62±0.05 m, mass=54.1±2.2 kg, 100 m PB=11.79±0.24 s) - classified as highly trained (14), elite (5) and world class (2) according to McKay and colleagues' framework (McKay et al., 2021) - volunteered for this study. Participants provided written informed consent after having the protocol explained to them, which was approved by the institutional research ethics committee (612/2020) and completed in accordance with the Declaration of Helsinki.

### Protocol

Sprints were performed at an outdoor athletics stadium during routine training sessions in the competition phase of the season, where training regularly included block starts. Participants completed their habitual warm up, which included multiple sub-maximal block starts. After warm up, participants performed three maximal effort trials of at least 20 m from blocks, separated by at least 5 min rest. Participants used their own spikes and preferred block settings.

### Data collection

**Fig. 5.**
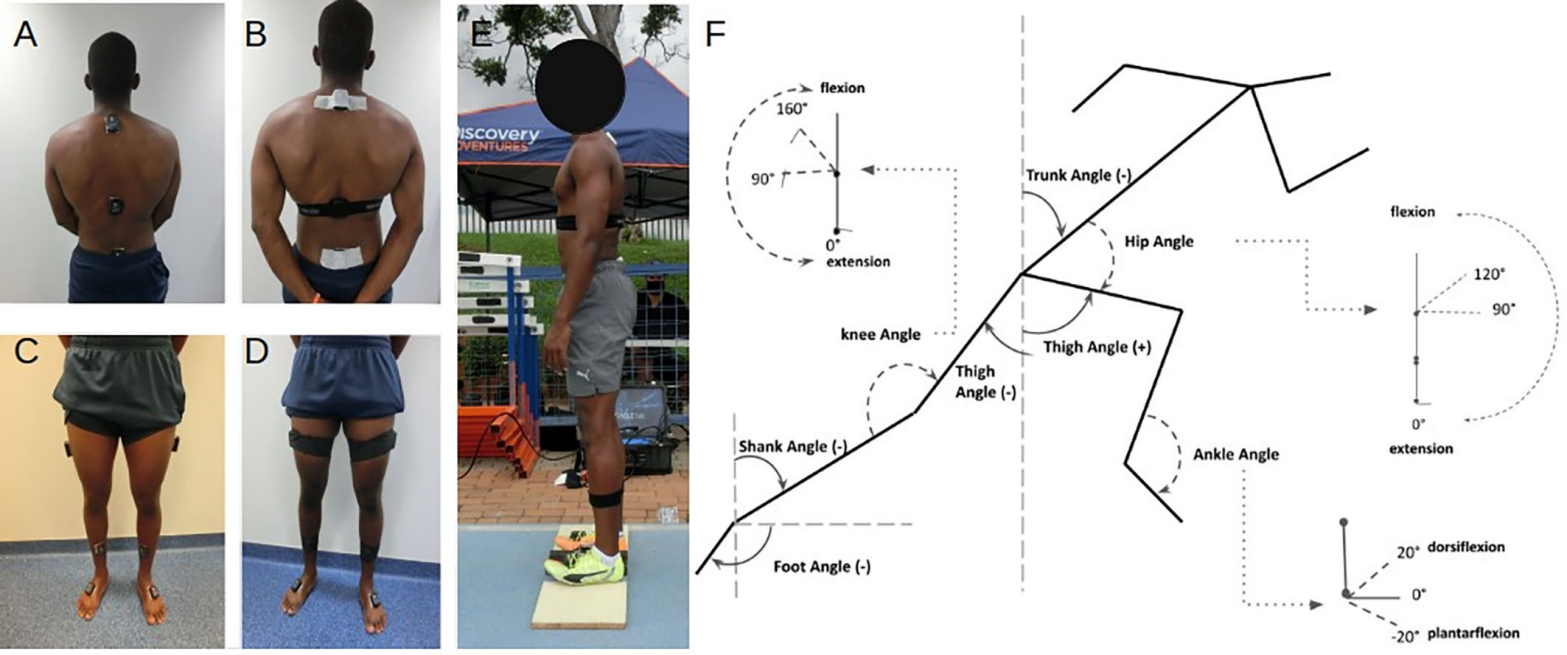
**Upper body (A and B) and lower body (C and D) IMU sensor placements and attachments.** Calibration posture with full sensor setup (E) and segment and joint definitions used in this study (F).

Instantaneous velocity was recorded using a radar gun (47 Hz; Stalker Pro II ATS, Stalker, USA) from which split times were derived (Samozino et al., 2016). Three-dimensional (3D) kinematics were recorded using tri-axial inertial measurement units (IMU) (200 Hz, MyoMotion; Noraxon, USA), for which sagittal plane validity and reliability has previously been reported (Berner et al., 2020; Balasubramanian, 2013; Yoon, 2017; Cottam et al., 2022) and which have been used in previous sprint research (e.g. Struzik et al., 2015, 2016; Bayne et al., 2020). Between warm up and sprint trials, participants were fitted with nine IMU sensors, affixed to the upper spine (T1), lower spine (T12) and sacrum as well as the lateral aspect of the left and right thigh, medial aspect of each shin and dorsal surface of each foot (Fig. 5A-E). Upper spine and pelvis sensors were secured using double sided tape, after the area had been towelled dry and prepared using alcohol swabs and an adhesive spray. Adhesive tape was then applied over the sensors (Fig. 5B). The lower spine sensor was attached using a manufacturer-supplied custom Velcro strap (Fig. 5B), applied tightly to avoid moving or slipping due to impact or sweat, but not so tight that it restricted breathing. Thigh and shank sensors were attached using double sided tape (Fig. 5C) and secured tightly with self-adhesive bandages (Fig. 5D) so as to minimise movement due to soft-tissue artifact or impact. Foot sensors were attached in manufacturer provided plastic clips on the upper portion of the foot and the laces pulled tight over the sensor, through the available hooks in the clip, and tape applied over the laces. Sensors were thus securely attached and checked before each trial. Trials where a sensor came loose were excluded.

Sagittal plane video of the first four steps after block exit was recorded by a camera (120 Hz, Ninox-250, Noraxon, USA) placed perpendicular to the recording lane, at a distance of 5 m (approximate field of view=6 m wide). IMU data and video were synchronised and captured using MyoResearch 3.14 software (Noraxon, USA).

### Data processing

Segment kinematics were obtained from a 9-segment rigid-body model included in the IMU manufacturer's software (MyoResearch 3.14). IMU sensors were calibrated prior to each trial to establish the local coordinate system. The IMU system establishes a 0^°^ reference angle for segment orientations in all planes during calibration, from which subsequent kinematic measurements are based. Within-participant reliability in the calibration position has been demonstrated, given standardised instructions (Berner et al., 2020; Donaldson et al., 2021). Participants stood in a neutral upright posture on a calibration board fixed with guides to set the feet in parallel, at approximately hip width for each participant. Participants were instructed to “maintain an upright, neutral posture with hands placed at the sides and head looking forward” and remained in this position until calibration was complete (Fig. 5E).

Magnetometer, gyroscope and accelerometer signals were fused during capture using a Kalman filter fusion algorithm applied by the software. A light anti-wobble filter was applied within the MyoResearch software to reduce potential soft-tissue artifact in the signal. The anti-wobble filter used a spherical linear interpolation (SLERP, 300 ms) and a low pass butterworth filter (15 Hz) to smooth IMU signal.

Toe off (TO) and touchdown (TD) were identified from sagittal plane video. Touchdown was determined as the first frame with visible ground contact and toe off as the first frame in which the foot no longer visibly contacted the ground. Steps were defined from toe off to the next toe off of the contralateral leg, beginning with front-foot block clearance (TO_0_). Therefore, flight time was defined as the time from toe off of one step until touchdown of the contralateral leg in the next step, such that flight time for step 1 represented the time from block clearance (TO_0_) to touchdown in step 1 (TD_1_). Contact time was defined as time between touchdown and toe off in the same step. From IMU data, sagittal plane angles for the trunk, thigh, shank and foot segments as well as the hip, knee and ankle joints were normalized to 101 data points for each step. Trunk orientation was determined from the upper spine sensor (T1). Angle definitions are presented in Fig. 5F. Segment rotations were described as clockwise or anticlockwise relative to a left-to-right direction of motion (Fig. 6A). Limbs were classified as ‘leading’ or ‘trailing’ based on their relative position at each toe off, such that the swing leg at toe-off was considered ‘leading’ and stance leg ‘trailing’ for the duration of the subsequent step. As such, since the limbs' oscillatory motion during running, ‘leading’ limb at toe off in step 1 became ‘trailing’ limb at toe-off instep 2 and vice versa (Fig. 6B).

### Coordination analysis

**Fig. 7.**
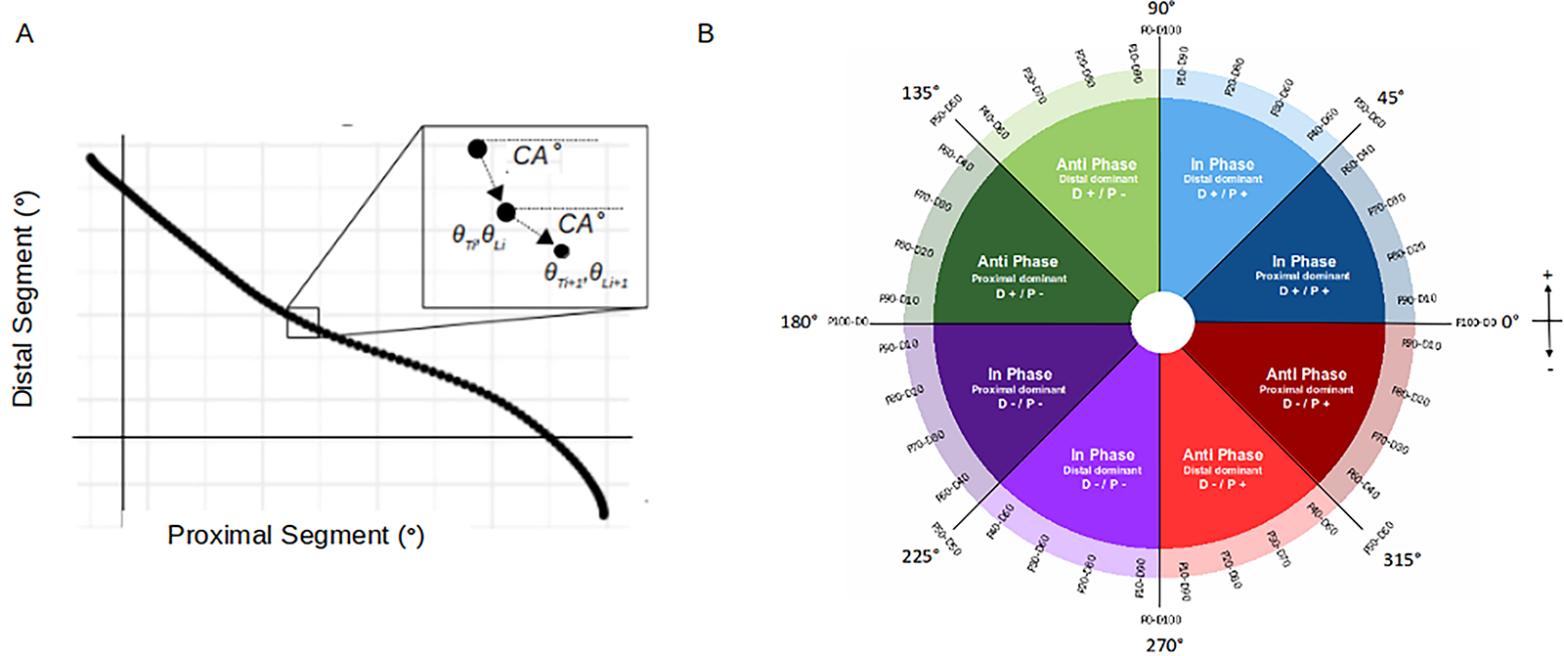
**(A) Angle-angle plot with coupling angle definition.** (B) Coordination bins and segment dominancy conventions for general proximal and distal segment couplings, adapted from Needham et al. (Needham et al., 2020).

Coupling angle mapping was used to profile individual coordination over the first four steps (Needham et al., 2014, 2020). Thigh-thigh, trunk-shank, and shank-foot segment couplings were assessed with a proximal-distal naming convention. For the thigh-thigh coupling, the trailing and leading thigh were designated as proximal and distal respectively. Coupling angles (CA) were calculated from angle-angle plots of segment couplings using a modified vector coding approach (Chang et al., 2008; Needham et al., 2014, 2020). The CA represented the vector angle between adjacent points in the angle-angle diagram relative to the right horizontal, expressed as an angle between 0° and 360° (Fig. 7A). Thus, for each normalised time point, CA position on the circular plane described the relative rotation of the two segments (Fig. 7B). For any two segments, rotation could either be in-phase (same direction) or anti-phase (opposite direction) and segments could either rotate clockwise or anticlockwise. Consequently, there were four possible relationships between segments, corresponding to the circular plane's four quadrants. Each quadrant was further divided into two 45° bins based on the dominant segment, i.e. which segment underwent the greatest rotation in a given time period, resulting in eight distinct coordination bins describing the relationship between segment rotations (in-phase or anti-phase), the direction and the dominant segment (Needham et al., 2020; Bezodis et al., 2019a) (Fig. 7B). The magnitude of segment dominancy (i.e. which segment underwent greater rotation) was quantified according to Needham et al. (2020). Briefly, since 90° is equal to 100 gradians, each circular plane quadrant can be represented as 0 to 100%. Converting the CA to gradians gives the proximal or distal segment dominancy as a percentage at every normalised time point (Needham et al., 2020) (Fig. 7B). For example, a 90° CA equals 100 gradians, and therefore 100% segment dominancy. A 100% dominant proximal segment reflects a rotating proximal segment and a completely fixed distal segment, for that time period, while 50% segment dominancy reflects equal rotation. Since bins were defined according to dominant segment and dominant segment switches as 50% mark is crossed, segment dominancy was constrained between 50% and 100%. Primary coordination patterns were classified by colour, and distal or proximal segment dominancy illustrated by light or dark shades of each colour, respectively. Therefore, changes between colours represented overall coordination changes and changes in tone within a given colour represented change in dominant segment. Individual coordination was profiled by plotting the segment dominancy over time and each bar colour coded by coordination bin, as determined by CA position on the circular plane (Needham et al., 2020).

### Data analysis

Mean coordination profiles were determined using circular statistics (Needham et al., 2020; Chang et al., 2008). Between-individual variation in coordination was evaluated from the standard deviation at each time point (Needham et al., 2020). Specific between-individual differences in coordination patterns were identified by visually inspecting coordination profiles. Step-to-step coordination changes were assessed using a coupling angle difference score (CA_Diff_) (Bezodis et al., 2019a; Brazil et al., 2020). Briefly, the coordination bin at each normalised time point was compared to the corresponding point in the subsequent step and assigned a score between 0 (same bin) and 4 (opposite bin). The total sum of difference scores over the entire step was represented as a percentage of the maximal possible score. A lower CA_Diff_ indicated more similar coordination (Bezodis et al., 2019a). Further, the frequency of each bin was compared across steps using non-parametric Friedman's tests and pairwise differences assessed using Wilcoxon signed-rank tests. Touchdown and toe off angular kinematics were assessed with one-way repeated measures analyses of variance (ANOVA) and pairwise *t*-tests. All pairwise tests were adjusted for multiple comparisons with a Bonferroni correction. For ANOVAs, sphericity assumptions were assessed with Mauchly's tests and a Greenhouse-Geisser corrections applied to variables that violated the assumption. All tests were performed in R (R Core Team, 2020) using the *rstatix* package (Kassambara, 2021). Alpha level was set at 0.05.
